# Efficacious Web-Based Psychotherapy to Address Depression and Anxiety Among Patients Receiving Oncological and Palliative Care: an Open-Label Randomised Controlled Trial

**DOI:** 10.1192/j.eurpsy.2023.1032

**Published:** 2023-07-19

**Authors:** N. Alavi, M. Omrani, A. Shirazi, G. Layzell, J. Eadie, J. Jagayat, C. Stephenson, D. Kain, C. Soares, M. Yang

**Affiliations:** 1 Psychiatry, Queen’s University, Kingston, Canada; 2 Psychiatry, OPTT inc, New York, United States; 3 OPTT inc, Toronto; 4 neuroscience; 5 Queen’s University, Kingston, Canada

## Abstract

**Introduction:**

Oncological and palliative care patients face unique stressors which increase their risk of developing depression and anxiety. Cognitive behavioural therapy (CBT) and mindfulness has established success in improving this population’s mental health. Traditional face-to-face psychotherapy is costly, has long wait lists, often lacks accessibility, and has strict scheduling, each of which can make attending psychotherapy physically, mentally, and financially out of reach for oncological and palliative patients. Web-based CBT (e-CBT) is a promising alternative that has shown efficacy in this and other patient populations.

**Objectives:**

To quantify the efficacy of online CBT and mindfulness therapy in oncological and palliative patients experiencing depression and anxiety symptoms.

**Methods:**

Participants with depression or anxiety related to their diagnosis were recruited from care settings in Kingston, Ontario, and randomly assigned to 8 weekly e-CBT/mindfulness modules (N= 25) or treatment as usual (TAU; N=24). Modules consisted of CBT concepts, problem-solving, mindfulness, homework, and personalised feedback from their therapist through a secure platform (Online Psychotherapy Tool- OPTT) Participants completed PHQ-9 and GAD-7 in weeks 1, 4, and 8. (NCT04664270: REB# 6031471).

**Results:**

Significant decreases in PHQ-9 and GAD-7 scores within individuals support the hypothesis of efficacy. At this time, 10 e-CBT/mindfulness and 12 TAU have completed the study. Decreases in PHQ-9 and GAD-7 scores within e-CBT group support the hypothesis of efficacy. Specifically, PHQ-9 scores decreased over the 3 repeated measures (ANOVA, 2 groups, 3 repeated measures and the decrease in GAD-7 scores was similarly large)

**Conclusions:**

As hypothesized, the results suggest that e-CBT/mindfulness therapy is an affordable, accessible, and efficacious mental health treatment for this population. The virtual, asynchronous delivery format is particularly appropriate given the unique barriers.

**Disclosure of Interest:**

N. Alavi Shareolder of: OPTT inc, Grant / Research support from: department psychiatry Queen’s University, M. Omrani Shareolder of: OPTT inc, A. Shirazi: None Declared, G. Layzell: None Declared, J. Eadie: None Declared, J. Jagayat: None Declared, C. Stephenson: None Declared, D. Kain: None Declared, C. Soares: None Declared, M. Yang: None Declared

